# LONGITUDINAL FACTORS ASSOCIATED WITH LIVING WELL IN PEOPLE WITH MILD TO MODERATE DEMENTIA

**DOI:** 10.1093/geroni/igad104.1429

**Published:** 2023-12-21

**Authors:** Linda Clare, Laura Gamble, Anthony Martyr, Fiona Matthews

**Affiliations:** University of Exeter Medical School, Exeter, England, United Kingdom; Newcastle University, Newcastle upon Tyne, England, United Kingdom; University of Exeter, Exeter, England, United Kingdom; Newcastle University, Newcastle upon Tyne, England, United Kingdom

## Abstract

Understanding the factors associated with quality of life can help us become more effective in optimizing the potential for people with mild-to-moderate dementia to ‘live well’. Identifying individuals at risk of declining quality of life could help to target support more appropriately. We used longitudinal data from 1749 individuals with mild-to-moderate dementia participating in the IDEAL cohort. Assessments took place on entry and at 12, 24, 48, 72 and 96 months. Using mixed effects modelling we examined change in quality of life, as measured by QoL-AD, since time of diagnosis, and looked at time-invariant and time-varying measures and their association with decline in quality of life at 6 years. Mean quality of scores life were stable over our six year follow up period. However, we were able to identify some individuals whose quality of life declined by varying amounts over this period. A larger decline was associated with being male and older, and those with a decline in quality of life had worsening cognitive function and increasing depression in the years following diagnosis. Our results show that quality of life does not decline for most people following a diagnosis of dementia. However, for those that did decline we were able to identify key factors associated with this decline. This model allows us to investigate further factors that may influence living well in people with dementia, and identify, at diagnosis, those most ‘at risk’ of decline and identify potential remediating factors that could inform potential intervention to mitigate this decline.

